# Gene polymorphisms of miR-323b and miR-1343 that regulate kininogen L are associated with schizophrenia susceptibility: A preliminary population‑based study

**DOI:** 10.17305/bb.2024.11100

**Published:** 2024-10-25

**Authors:** Xiaoyu Liu, Mengdi Jin, Mingjia Yang, Lijuan Yan, Weijiao Zhao, Lizhuo Liu, Hongmin Wang, Yongzhuo Ding, Yanyan Sun, Yanchi Zhang, Qiong Yu

**Affiliations:** 1 Department of Psychology, Changchun Sixth Hospital, Changchun, China; 2 Department of Epidemiology and Biostatistics, School of Public Health, Jilin University, Changchun, China

**Keywords:** Kininogen-L, miR-SNP, schizophrenia, auditory hallucinations

## Abstract

miRNA-related single-nucleotide polymorphism (miR-SNP) is a type of functional SNP that affects the regulatory functions of miRNA genes, miRNA binding sites, or components of miRNA biogenesis. This study aimed to explore the relationship between miRNA gene polymorphisms that regulate the kininogen L protein and schizophrenia (SCZ). Bioinformatics methods predicted miRNA gene polymorphism sites regulating the kininogen L protein. The polymorphisms of rs56103835, rs6513496, rs651349, and rs2986407 were detected using improved multiple ligase detection reaction multiple SNP typing technologies in 513 SCZ patients and 509 controls. The association of miR-SNP variations with SCZ susceptibility and symptoms was evaluated using SNPstat to determine the optimal inheritance model. Generalized multifactor dimensionality reduction analysis and logistic regression were used to calculate miR-SNP interactions. The association between hsa-miR-323b-rs56103835 and SCZ was statistically significant under the dominant model. The result of gene–gene interaction showed that the three-factor model (rs56103835/rs2986407/rs2155248) was the best, but it could not be considered significantly related to SCZ. Additionally, SCZ patients with the CC or CT genotype on rs2986407 were more likely to experience auditory hallucinations than those with the TT genotype. Our data revealed that the mutation of hsa-miR-323b-rs56103835 from C to T was associated with susceptibility to SCZ. The mutation of hsa-miR-1343-rs2986407 from T to C increases the risk of auditory hallucinations in SCZ patients.

## Introduction

Schizophrenia (SCZ) is a persistent, serious mental disorder that affects approximately 1% of the global population [1]. This condition is causally linked to high levels of morbidity, imposing significant personal and societal costs [2–4]. SCZ symptoms are diverse, encompassing positive symptoms (e.g., hallucinations and thought disorders), negative symptoms (e.g., social withdrawal and emotional blunting), and other psychopathological manifestations such as lack of insight and impulse control [5]. The epidemiology of SCZ shows considerable variability, with incidence and prevalence rates influenced by geographical, socioeconomic, and other contextual factors [6]. Although the exact cause of SCZ remains unclear, it is widely acknowledged that both genetic and environmental factors play roles in its development. Recent studies suggest a heritability of around 79% for SCZ [7]. miRNAs, brain-enriched non-coding RNAs, are approximately 22 nucleotides in length [8, 9]. They play multiple regulatory roles in cells, combining with complementary sequences to suppress translation and degrade target mRNAs [8–10]. miRNAs are estimated to influence about one-third of human genes [11]. Their structure is highly conserved, demonstrating significant sequence homology and consistency in gene location. Although miRNAs are subject to transcriptional regulation and cis-acting gene mutations [12], they are also extensively controlled at the post-transcriptional level and are sensitive to the pathways involved in their biosynthesis [13]. Increasing evidence indicates that variations in miRNA processing and maturation may contribute to the onset and progression of various pathophysiological conditions, including neurological and neuropsychiatric disorders like SCZ. In this context, copy number variations (CNVs) and gene polymorphisms in miRNA biosynthetic pathways are frequently highlighted in SCZ and related neurobehavioral disorders. Single-nucleotide polymorphisms (SNPs), the most common form of genetic variation, result from single-nucleotide changes in the genome. They account for over 90% of all known human polymorphisms. In 2014, the Schizophrenia Working Group of the Psychiatric Genomics Consortium conducted a large-scale genome-wide association study (GWAS) for SCZ, identifying 108 genetic loci significantly associated with the disorder. Subsequent large-scale sequencing, linkage studies, and candidate gene research have continued to identify new genetic associations. For example, Heidari Nia et al. [14] found that *GABRB2* polymorphisms were linked to an increased risk of SCZ in an Iranian population. Similarly, Sargazi et al. [15] identified that the rs12407427 T/C variant in KIF26B could serve as a novel genetic biomarker for SCZ in Iranian samples. PPARG rs1821282 and rs3856806 polymorphisms have also been associated with SCZ susceptibility [16]. miRNA-related SNPs (miR-SNPs), which are functional SNPs in miRNA genes, their binding sites, or biogenesis mechanisms, can alter miRNA expression and function. Variations in miRNA genes or their processing components can impair miRNA synthesis by altering their sequence or processing. SNPs in the binding sites of target genes can influence the interaction between miRNAs and their targets [17, 18]. This emerging class of SNPs offers new avenues for SCZ biomarker research. Furthermore, miR-SNPs tend to be highly conserved, passing on to future generations in a stable manner. These variations are key indicators of individual diversity, influencing traits, phenotypes, and disease progression. Hansen’s pioneering research in this field revealed that rs1700 in miR-198 and rs17578796 in miR-206 are associated with SCZ [19]. Subsequent studies have reported associations between SCZ and SNPs in miR-498 [20], pre-miR-30e [21], and miR-143 [22] across various populations. Previous proteomic studies identified kininogen L as a potential protein marker for SCZ, with significant associations between SCZ and Cystatin 9 (*CST9)* gene polymorphisms that encode kininogen L [23]. Additionally, studies have confirmed that variations in miRNA sequences can impact their function in SCZ patients [24]. Based on these findings, this study aims to explore the role of miRNA-SNPs in SCZ by examining how these polymorphisms alter the regulation of the target gene *CST9* by miRNAs.

## Materials and methods

### Participants

In this study, based on SNP gene frequency, the prevalence rate of SCZ (1%), and the estimated genetic risk (1.4–2.0) [25], the sample size and power analysis were conducted using Quanto software. A minimum sample size of 971 subjects was determined to achieve a statistical power of 0.85. To account for potential sample loss during genotype detection, a total of 1022 participants were recruited for this case-control study. The cohort included 513 SCZ patients from Changchun Sixth Hospital and 509 healthy controls (CTLs) enrolled during the same period. Clinical diagnoses were confirmed by at least two consultant psychiatrists based on the Tenth Revision of the International Classification of Diseases (ICD-10) criteria [26]. All patients were of Han descent from northern China, with exclusion criteria, including other severe medical diseases, major psychiatric disorders, structural brain abnormalities, brain trauma, and movement disorders. Control participants had no family history of mental illness, no personal history of mental disorders, and no blood transfusions in the three months preceding the study. SCZ and CTL groups were matched for gender, age, and ethnicity.

### miRNA prediction and SNP selection

In prior research, kininogen L was identified as a potential protein marker for SCZ, with *CST9* gene polymorphisms showing significant associations with SCZ susceptibility. Using TargetScan [27], PITA [28], and DIANA-micro T [29], miR-323b, miR-646, miR-1343, and miR-646 were predicted as miRNAs regulating CST9. RNAhybrid was employed to assess the interaction strength between *CST9* and these miRNAs, based on total free energy [30]. SNPs with a minor allele frequency (MAF) > 10% in the Han population of northern China (CHB) were retrieved from the NCBI dbSNP database. Four SNPs—rs56103835, rs6513497, rs2986407, and rs6513496—were selected for further analysis, as shown in Table 1.

**Table 1:** Information of miRNA prediction and SNP selection

**Software**	**Target gene**	**Putative miRNA**	**SNP**	**Position (GRCh38.p14)**	**MAF**
TargetScan	*CST9*	hsa-miR-323b	rs56103835	chr14:101056219	0.2979
PITA	*CST9*	hsa-miR-646	rs6513497	chr20:60308547	0.1909
DIANA-microT	*CST9*	hsa-miR-1343	rs2986407	chr11:34941869	0.2332
DIANA-microT	*CST9*	hsa-miR-646	rs6513496	chr20:60308476	0.2728

MAF: Minor allele frequency; SNP: Single-nucleotide polymorphisms; CST9: Cystatin 9.

### DNA extraction and SNP genotyping

Genomic DNA was extracted from whole blood using the Whole Blood DNA Extraction Kit (Abigen, Beijing). In brief, protein precipitation was added after cell dissolution with a red blood cell lysis solution. DNA was then precipitated from the supernatant using isopropyl alcohol, washed twice with 70% ethanol, and stored at –80 ^∘^C for genotyping. DNA concentration and purity were measured with a nucleic acid protein analyzer (BIOSPEC-NANO, Japan), with DNA concentrations consistently above 10 ng/µL and an OD260/280 ratio between 1.8 and 2.0. SNP genotyping was conducted using the improved multiple ligase detection reaction (iMLDR), in collaboration with the Center for Human Genetics Research, Shanghai Genesky Biotechnology Company.

The target SNP segment was amplified in a 10 µL PCR reaction containing Taq polymerase, dNTPs, MgCl2, PCR buffer (Qiagen Inc.), 1 µL template DNA, and 1 µL primers (see Table S1 for PCR primer sequences). The PCR program consisted of an initial 95 ^∘^C for 2 min, followed by 35 cycles of 94 ^∘^C for 20 s, 65 ^∘^C for 40 s, and 72 ^∘^C for 90 s, and 24 cycles of 94 ^∘^C for 20 s, 59 ^∘^C for 30 s, and 72 ^∘^C for 90 s, ending with a final extension at 72 ^∘^C for 2 min. The amplified product was purified with ExoI/SAP enzymes and used as the template for ligation. Each SNP site was then ligated with two 5′ allele-specific probes and a 3′ fluorescently-labeled probe in a ligation reaction containing ligase buffer, ligase, 2 µL PCR product, ddH2O, and ligation primers (see Table S2 for primer sequences). The ligation procedure consisted of 38 cycles at 94 ^∘^C for 1 min and 56 ^∘^C for 4 min. A 0.5 µl Liz500 Size Standard and 9 µL Hi-Di were added to 0.5 µL of the diluted ligation product, which was denatured at 95 ^∘^C for 5 min and analyzed by capillary electrophoresis on an ABI 3730XL. Raw data files were processed using GeneMapper 4.1 software.

### Temporal and spatial expression patterns of miRNA genes in the human brain

To investigate the temporal and spatial expression patterns of SNPs in the human brain, gene expression data were downloaded from the PsychENCODE database, which contains 1866 samples of postmortem brain tissue. The study analyzed the expression of miR-SNP-localized genes in different brain regions (neocortex, hippocampus, amygdala, striatum, cerebellar cortex, and medulla) from gestational day 50 through the 10th postnatal day, to explore potential involvement in the onset and progression of SCZ and its symptoms.

### Ethical statement

Approval was granted by the Ethics Committee of the School of Public Health, Jilin University (2016-03-013), and all participants provided written informed consent.

### Statistical analysis

Demographic characteristics and clinical symptoms between cases and controls were compared using *χ*^2^ and rank-sum tests. The Hardy–Weinberg equilibrium (HWE) test was conducted using a goodness-of-fit test. The allele frequency and genotype distribution of miRNA-SNPs were analyzed with a *χ*^2^ test, and the optimal inheritance model for each SNP was determined based on the Akaike information criterion (AIC) calculated by SNPstats (https://www.snpstats.net/snpstats/). Gene interactions were evaluated using generalized multifactor dimensionality reduction (GMDR) software and logistic regression analysis. Statistical analyses were conducted with SPSS version 24.0, with significance set at *P* < 0.05.

## Results

### Characteristics of participants and HWE

A total of 1022 participants were included in the study, with 513 in the SCZ group (273 males and 240 females; median age: 30, range: 24–39) and 509 in the CTL group (244 males and 265 females; median age: 30, range: 24–37). No significant differences in age (*Z* ═ −0.403, *P* ═ 0.687) or gender (*χ*^2^ ═ 2.849, *P* ═ 0.091) were found between the SCZ and CTL groups. All SNPs were in HWE (*P* > 0.05), except for rs2986407 in the control group (*P* ═ 0.041).

### Allele and genotype frequency distribution of miR-SNPs

The detection rates of alleles and genotypes for rs56103835, rs6513497, rs2986407, and rs6513496 in the SCZ group were 100%, 100%, 99.6%, and 100%, respectively. In the control group, the detection rates were 99.6%, 100%, 95.9%, and 100%, respectively. No significant differences in allele or genotype frequency distributions were observed at these loci (*P* > 0.05). See Table 2 for details.

**Table 2:** Allele frequency and genotype distribution in case and control groups (%)

**Allele/Genotype**	**SCZ (*n* ═ 513)**	**Control (*n* ═ 509)**	***χ^2^***	***P***
*rs56103835*				
C	731 (71.0)	687 (68.0)	2.94	0.086
T	295 (29.0)	327 (32.0)		
CC	269 (52.4)	233 (46.0)	4.48	0.107
CT	193 (37.6)	221 (43.6)		
TT	51 (10.0)	53 (10.4)		
*rs6513497*				
T	920 (90.0)	921 (90.0)	0.37	0.544
G	106 (10.0)	97 (10.0)		
TT	409 (79.7)	414 (81.3)	–	0.806^*^
TG	102 (19.9)	93 (18.3)		
GG	2 (0.4)	2 (0.4)		
*rs2986407*				
C	796 (78.0)	751 (77.0)	0.25	0.615
T	226 (22.0)	225 (23.0)		
CC	311 (60.9)	297 (60.9)	1.73	0.420
CT	174 (34.0)	157 (32.2)		
TT	26 (5.1)	34 (6.9)		
*rs6513496*				
T	894 (87.0)	904 (89.0)	1.34	0.247
C	132 (13.0)	114 (11.0)		
TT	390 (76.0)	401 (78.8)	1.40	0.496
TC	114 (22.0)	102 (20.0)		
CC	9 (2.0)	6 (1.2)		

^*^Fisher’s exact test. SCZ: Schizophrenia.

### Association of miR-323b-rs56103835 with SCZ susceptibility

The rs56103835 polymorphism was significantly associated with SCZ susceptibility under the dominant model (*P* ═ 0.038). Individuals with CT and TT genotypes had a lower risk of SCZ compared to those with the CC genotype (OR ═ 0.77, 95% CI: 0.60–0.99, *P* ═ 0.038). See Table 3 for details. The inheritance models for rs2986407, rs6513497, and rs6513496 were recessive, over-dominant, and dominant, respectively, but no significant associations were found between these SNPs and SCZ.

**Table 3:** The association between rs56103835 and schizophrenia

**Model**	**Genotype**	**SCZ (%)**	**Control (%)**	**OR (95% CI)**	***P***	**AIC**
Codominant	C/C	269 (52.4)	233 (46.0)	1.00	0.11	1415.5
	C/T	193 (37.6)	221 (43.6)	0.76 (0.58–0.98)		
	T/T	52 (9.9)	53 (10.4)	0.83 (0.55–1.27)		
Dominant	C/C	269 (52.4)	233 (46.0)	1.00	0.038*	1413.7
	C/T + T/T	244 (47.6)	274 (54.0)	0.77 (0.60–0.99)		
Recessive	C/C + C/T	462 (90.1)	454 (89.5)	1.00	0.79	1417.9
	T/T	51 (9.9)	53 (10.4)	0.95 (0.63–1.42)		
Over-dominant	C/C + T/T	320 (62.4)	286 (56.4)	1.00	0.052	1414.2
	C/T	193 (37.6)	221 (43.6)	0.78 (0.61–1.00)		

*P < 0.05. SCZ: Schizophrenia; OR: Odds ratio; AIC: Akaike information criterion.

### Interactions between miR-SNPs

**Figure 1.**
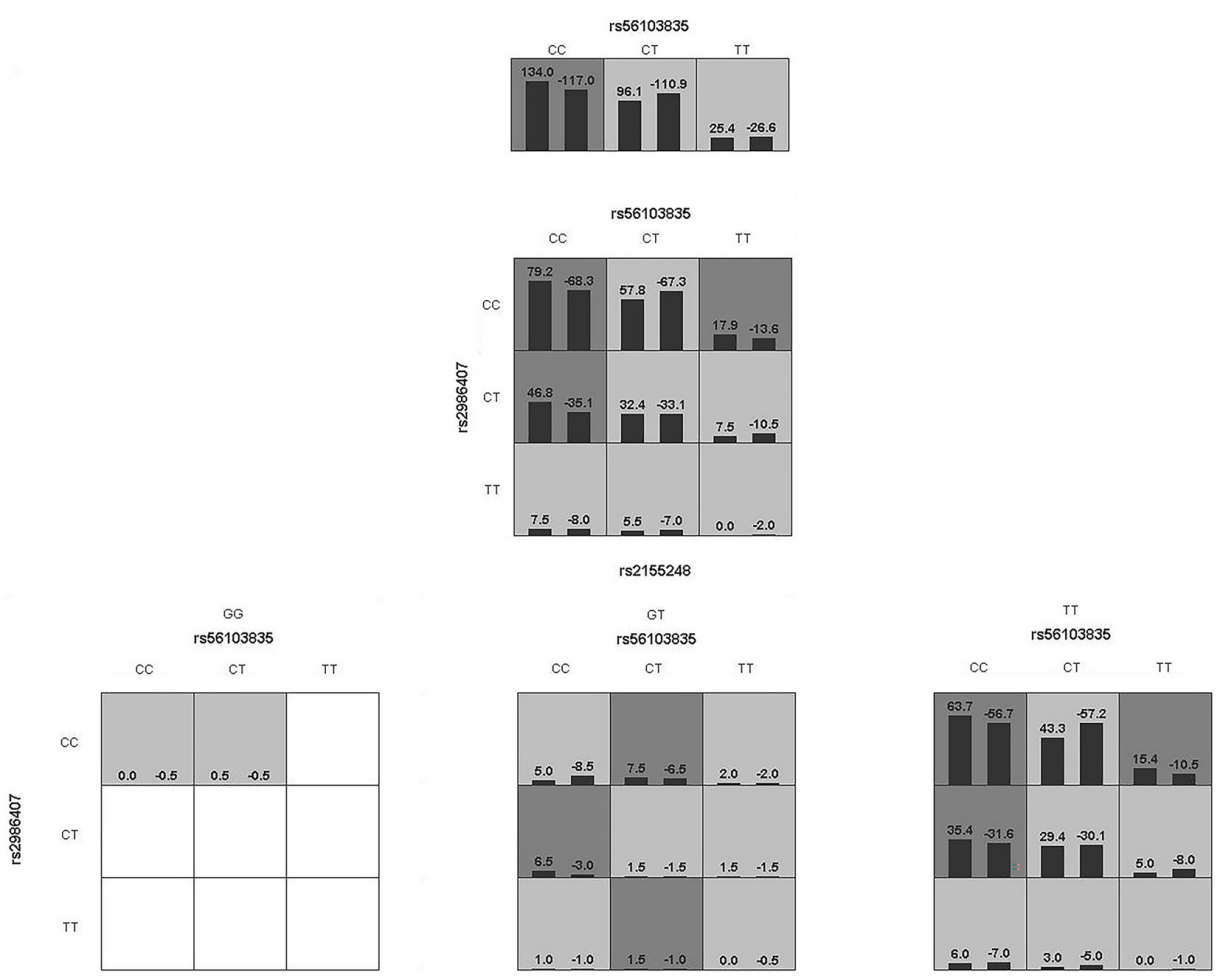
**A combined model of gene–gene interaction analysis.** The left bar of the cell represents the SCZ patients, the right one is the healthy control, the bar graph color indicates the high or low risk, and the blank cell indicates that there is no influence or no such genotype in the population. SCZ: Schizophrenia.

Logistic regression analysis revealed no SCZ-related interactions among the four miR-SNPs (see Table 4). GMDR analysis identified a three-factor model (rs56103835-rs2986407-rs2155248) as the best model, with a testing-balanced accuracy of 53.62% and the highest cross-validation consistency (10/10) (Table 5). However, this model was not significantly associated with SCZ risk (*P* ═ 0.1719). The specific interaction model is shown in Figure 1.

**Table 4:** Logistic regression analysis of miR-SNP interactions

**Regression model**	**B**	**S.E.**	**Wald**	***P***	**OR (95% CI)**
rs56103835*rs6513496	0.193	0.200	0.928	0.335	1.213 (0.819, 1.795)
rs2986407*rs56103835	0.554	0.388	2.041	0.153	1.741 (0.814, 3.725)
rs2986407* rs6513496	0.543	0.454	1.430	0.232	1.721 (0.707, 4.189)
rs2986407*rs6513497	−0.046	0.063	0.529	0.467	0.955 (0.547, 1.612)

B: Regression coefficients; S.E: Standard error; Wald: Chi-square value; OR: Odds ratio; miR-SNP: miRNA-related single-nucleotide polymorphism.

**Table 5:** Summary of GMDR results of gene–gene interaction

**Model**	**Tr-BA**	**Te-BA**	***P***	**CVC**
rs56103835	0.5340	0.5172	0.1719	9/10
rs56103835-rs2986407	0.5540	0.5355	0.1719	10/10
rs56103835-rs2986407-rs2155248	0.5586	0.5362	0.1719	10/10

Tr-BA: Training balanced accuracy; Te-BA: Testing balanced accuracy; CVC: Cross-validation consistency; GMDR: Generalized multifactor dimensionality reduction.

### Association of miR-1343-rs2986407 with auditory hallucinations in SCZ patients

Clinically relevant information and symptoms of SCZ patients were also collected (Table 6). Under the recessive model for rs2986407, patients with the TT genotype were 2.704 times more likely to experience auditory hallucinations compared to patients with the CC or CT genotypes (OR ═ 2.704, 95% CI: 1.204–6.075, *P* ═ 0.013).

**Table 6:** Association of rs2986407 genotype and SCZ-related symptoms

**Hallucination symptoms**		**Genotype**			
		**TT**	**CC+CT**	**OR (95% CI)**	***P***
Auditory hallucination	+	13	137	2.704 (1.204, 6.075)	0.013*
	−	12	342		
Flavor hallucination	+	1	7	2.810 (0.332, 23.761)	0.336^a^
	−	24	472		
Murdered delusion	+	6	127	0.875 (0.342, 2.241)	0.781
	−	19	352		
Influence delusion	+	17	344	0.834 (0.352, 1.978)	0.680
	−	8	135		
Jealous delusion	+	18	322	1.254 (0.513, 3.064)	0.619
	−	7	157		
Self-blame delusion	+	18	372	0.740 (0.301, 1.818)	0.509
	−	7	107		
Thinking disorder	+	14	259	1.081 (0.481, 2.430)	0.850
	−	11	220		
Illusion delusional syndrome	+	6	133	0.822 (0.321, 2.102)	0.681
	−	19	346		

a: Fisher’s exact test; *P < 0.05. SCZ: Schizophrenia.

### Temporal and spatial expression patterns of miR-323b and miR-1343 in the human brain

**Figure 2.**
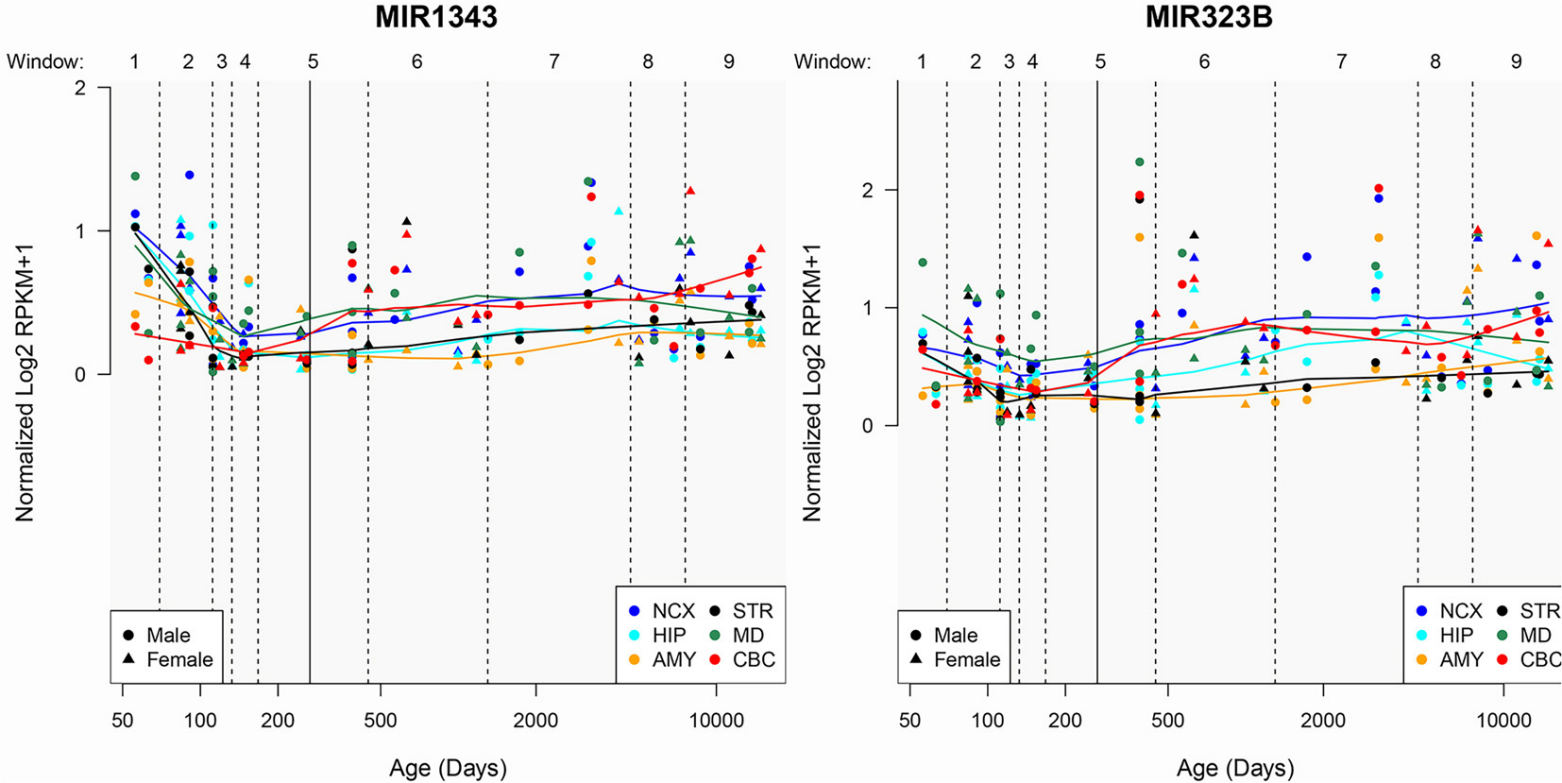
**The expression levels of miR-323b and miR-1343 in different brain regions changed with the development of the human brain.** NCX: Neocortex; HIP: Hippocampus; AMY: Amygdala; STR: Striatum; MD: Medulla; CBC: Cerebellar cortex.

This study found that the rs56103835 mutation increases susceptibility to SCZ, while the rs2986407 mutation raises the risk of auditory hallucinations in SCZ patients. rs56103835 and rs2986407 are single-nucleotide variations in miR-323b and miR-1343, respectively. To further explore their temporal and spatial expression patterns in the human brain, we used the PsychENCODE database to analyze changes in miR-323b and miR-1343 expression across various brain regions (neocortex, hippocampus, amygdala, striatum, cerebellar cortex, and medulla) from day 50 to day 10,000 post-birth (Figure 2).

It was observed that miR-1343 expression decreased significantly within the first 150 days after birth, with the most substantial decrease in the striatum, after which levels tended to stabilize. miR-1343 expression changes in other brain regions were relatively consistent, except in the cerebellar cortex, where levels continued to increase starting around day 150. miR-323b levels dropped significantly within the first 100 days of life, except in the amygdala. Afterward, expression levels in the neocortex and cerebellar cortex showed a continuous upward trend. These temporal changes in miR-323b and miR-1343 expression suggest their essential roles in early brain development.

## Discussion

In 1999, Irina first identified elevated plasma kallikrein–kinin system (KKS) activity in SCZ patients, while observing a significant reduction in brain kallikrein levels. This suggested that the KKS system may play a role in blood–brain barrier integrity and brain tissue damage, thereby influencing the onset and progression of SCZ [31]. Kininogen L, a crucial component of the KKS, interacts with factor XII, kallikrein, kinin, and kinases, and is involved in various physiological processes, such as nervous system regulation, thrombosis, inflammation, and apoptosis [32–34]. High-molecular-weight kininogen can be degraded into bradykinin by kallikrein, and bradykinin receptors are distributed throughout the central nervous system. Bradykinin promotes the synthesis and release of inflammatory mediators in the central nervous system and glial tissue, potentially leading to brain tissue damage or long-term disturbances in blood–brain barrier function [35]. In neurological diseases, such as Alzheimer’s disease and Parkinson’s disease, proteins involved in kinin production or kinin receptor function are often overexpressed [36]. *CST9* is a gene encoding kallikrein L, suggesting that miRNA-SNPs regulating *CST9* expression could influence kallikrein L levels and contribute to the development of SCZ. This study sought to explore associations between four miRNA-SNPs and SCZ.

One notable finding was that rs2986407 did not meet HWE in the healthy control group. The discussion around HWE has been ongoing; a population maintains HWE under five conditions: infinite size, random mating, no mutation, no migration, and no natural selection. However, in real populations, factors like inbreeding, selection, and mutation often disrupt HWE. Li [37] and Stark [38] both argue that random mating is a sufficient but non-essential condition for HWE, and Zou and Donner [39] showed that HWE testing is not always reliable for detecting allele-disease associations. Mayo noted that it is unrealistic to expect every SNP to meet HWE, especially for complex, multifactorial diseases; studying SNPs that deviate from HWE may be important in understanding disease mechanisms. For instance, in a case-control study of postpartum depression, Sun found that the T27224C genotype deviated from HWE (*P* < 0.05), yet significant genotype differences existed between case and control groups [40]. While some SNPs may not align with HWE, they can still play vital roles in disease development. After ruling out issues like sample size, genotyping error, and sample bias, rs2986407 was retained in this study for analysis.

A key finding of this study was the significant association of has-miR-323b rs56103835 with SCZ susceptibility under a dominant model. miR-323 is known to directly target the 3’-UTR of *BRI3* mRNA, a gene expressed predominantly in the brain. In ischemia-reperfusion contexts, increased miR-323 can inhibit BRI3 expression, reducing neuron survival and promoting apoptosis [41]. Although miR-323 has been infrequently studied in the context of mental illness, it has been shown to accelerate apoptosis induced by glucose deprivation and is linked to various neurodegenerative diseases like Alzheimer’s disease, Parkinson’s disease, and glioblastoma [42, 43]. A case-control study also found that rs56103835 was strongly associated with increased Alzheimer’s disease risk [44]. Therefore, the rs56103835 mutation may impact miR-323b expression levels, potentially influencing downstream apoptosis-related genes and contributing to SCZ pathology.

SCZ is a heterogeneous disorder, and susceptibility genes identified in case-control studies often show inconsistent associations. Different combinations of susceptibility genes may lead to distinct clinical subtypes of SCZ. SCZ symptoms can be divided into positive and negative categories, with hallucinations being a common positive symptom. Few genetic studies have examined specific symptoms in SCZ. Evidence suggests that the C allele of *CCK-AR* [45], the *FOXP241* polymorphism [46], and the *DTNBP1-*rs423616740 variant [47] are associated with a higher risk of auditory hallucinations in SCZ patients. This study is the first to show that the rs2986407 mutation is significantly associated with a higher incidence of auditory hallucinations in SCZ patients. Although the mechanism requires further investigation in animal models, these findings highlight the importance of studying symptom-based genetic sub-phenotypes of SCZ for treatment and patient management. For example, this finding could enable genetic screening and risk assessment, where SCZ patients with the rs2986407 mutation could be identified early and monitored more closely for auditory hallucinations. Patients carrying the rs2986407 mutation might benefit from medications that are particularly effective in treating auditory hallucinations, allowing for timely adjustments in treatment protocols.

Despite over two decades of genetic research on SCZ, the relationship between miR-SNPs and SCZ and its symptoms remains underexplored. Based on prior work, this study focused on the regulatory miRNA of *CST9*, which encodes Kininogen L. For the first time, we demonstrated an association between the miR-323b rs56103835 mutation and SCZ susceptibility, as well as the rs2986407 mutation and increased risk of auditory hallucinations in SCZ patients. This study has several limitations. First, uncertainties in bioinformatics predictions could affect validation outcomes. Second, we did not apply statistical correction due to the small sample size, which may have influenced our ability to detect true associations. Ideally, future studies should include the Positive and Negative Syndrome Scale (PANSS) scores to further refine symptom-specific associations with miR-SNPs. Although the mechanisms underlying SCZ and its psychiatric symptoms remain unclear, our work suggests that miR-SNPs are promising candidates for further investigation. Additional studies are needed to evaluate their functional and clinical significance in SCZ.

## Conclusion

The mutation of miR-323b-rs56103835 from C to T has been linked to an increased susceptibility to SCZ. The mutation miR-1343-rs2986407 from T to C has been associated with a heightened risk of auditory hallucinations in SCZ patients.

## Supplemental data

**Table S1:** Primers for the PCR

**SNPs**	**Primer sequence (5*′*-3*′*)**
rs56103835	F: GCCACCTGGTCCACTCATCCT
	R: TCGGCATCAGGTCCAAGAAGAC
rs6513497	F: CGGGTTTTCTCGAGCACTGTGT
	R: CCTGCATGCATTGGGGTAAAGA
rs6513496	F: CGGGTTTTCTCGAGCACTGTGT
	R: CCTGCATGCATTGGGGTAAAGA
rs2986407	F: CCCATCGGACAAAGGAACAGG
	R: AAAGAGATGGCAGCCCTGGAGT

SNP: Single-nucleotide polymorphism; F: Forward primer; R: Reverse primer.

**Table S2:** Primers for the ligation reaction

**SNPs**	**Primer sequence (5*′*-3*′*)**
rs56103835	FC: TCTCTCGGGTCAATTCGTCCTTTGCGAGCAGTGCCACCTGAC
	FT: TGTTCGTGGGCCGGATTAGT TGCGAGCAGTGCCACCTGAT
	FP: GGTACTCGGAGGGAGGTTGTCCTTTTTTTT
rs6513497	RG: TCTCTCGGGTCAATTCGTCCTTCCACTGAGCCTGAGGCCACC
	RT: TGTTCGTGGGCCGGATTAGTCCACTGAGCCTGAGGCCACA
	RP: GAGGCAGCTGCTTCCTCTCCTTTTTTT
rs6513496	RC: TCTCTCGGGTCAATTCGTCCTTTGACTCCACTGGCAGACTCCAGG
	RT: TGTTCGTGGGCCGGATTAGTTGACTCCACTGGCAGACTCCAGA
	RP: TCTCCAGGCTTCTGAACYGTCCTTTTTTTT
rs2986407	FC: TTCCGCGTTCGGACTGATATGGGGAGCGGCCCCCGTGC
	FT: TACGGTTATTCGGGCTCCTGTGGGGAGCGGCCCCCGTGT
	FP: GGGCCTCTGCTCTGGCCCTTTTTTTTTTT

F: Forward primer; R: Reverse primer; F and R indicate the orientation of the primer and the reference sequence. G, A, C, and T represent the nucleotide bases of the allele probe. P represents the universal primer for the site, used to initiate DNA amplification at that specific location. SNP: Single-nucleotide polymorphism.
